# Utilization and short-term outcomes of percutaneous left atrial appendage occlusion in patients with cancer

**DOI:** 10.1186/s40959-023-00192-z

**Published:** 2023-11-04

**Authors:** Yaqi Zhang, Zhuoran Yang, Muhammad U. Almani, Raquel Soon-Shiong, Bolun Liu

**Affiliations:** 1 Department of Internal Medicine, John H. Stroger, Jr. Hospital of Cook County, Chicago, IL USA; 2 Department of Internal Medicine, University of Iowa Hospitals and Clinics, Iowa City, IA USA; 3 Mount Sinai St Luke’s and Mount Sinai West Hospital, New York, NY USA; 4 Einstein Medical Center Philadelphia, Philadelphia, PA USA; 5 Mayo Clinic Health System, Mankato, MN USA

**Keywords:** Atrial fibrillation, Left atrial appendage occlusion, National inpatient sample, National readmissions database, Thirty-day readmission rate

## Abstract

**Background:**

Percutaneous left atrial appendage occlusion (LAAO) has been rapidly evolving since FDA’s approval in 2015 and has become more of a same-day-discharge procedure. Cancer patient with atrial fibrillation/flutter (AF) population can benefit from the procedure but the in-hospital outcomes and readmission data were rarely studied.

**Objectives:**

We investigated the utilization, in-hospital and readmission outcomes in cancer patients with AF who underwent LAAO.

**Methods:**

Data were derived from the National Inpatient Sample and National Readmissions Database from 2016 to 2019. Patients with primary diagnosis of AF admitted for LAAO (ICD-10 code 02L73DK) were grouped by cancer as a secondary diagnosis. We assessed in-hospital mortality, length of stay, total hospital charges, and complications. Thirty-day readmission rates were compared.

**Results:**

LAAO was performed in 60,380 patients with AF and 3% were cancer patients. There were no differences in in-hospital mortality and total hospital charges; however, cancer patients tended to have longer hospital stay (1.59 ± 0.11 vs. 1.32 ± 0.02, *p* = 0.013). Among complications, cancer patients had higher rates in open or percutaneous pericardial drainage (adjusted odds ratio [aOR] 2.38; 95% confidence interval [CI] 1.19–4.76) and major bleeding events (aOR 7.07; 95% CI 1.82–27.38). There was no statistical significance of 30-day readmission rates between patients with and without cancer (10.0% vs. 9.1%, *p* = 0.34). The most common readmission reason in cancer patients was gastrointestinal bleeding.

**Conclusions:**

LAAO is a promising procedure in cancer patients complicated by AF with contraindication to anticoagulation. Readmission rate is comparable between patients with and without cancer.

## Introduction

Atrial fibrillation/flutter (AF) is a common comorbidity in the patient population with cancer [1–4], though a causal relationship yet to be determined [5]. Patients with cancer face higher risk of thromboembolic complications [6, 7]; meanwhile, this population also has increased bleeding propensity [8]. New therapies in the field of cancer treatment are carrying cancer patients to an era of longer life expectancy, while aging is also a risk factor of AF [1, 2]. Anticoagulation in this specific patient group becomes an unavoidable clinical decision. Current guidelines mostly recommend low-molecular-weight heparin for thromboembolism prophylaxis in cancer patients with AF [9]; however, the bleeding propensity remains concerning [10].

Percutaneous left atrial appendage occlusion (LAAO) has become a “bypass” for anticoagulation in AF patients since the approval for the Watchman device (Boston Scientific Corp., Marlborough, MA, USA) in the United States in 2015. Thus, cancer patients with AF who have contraindications to anticoagulation could benefit from LAAO; however, scarce data were published regarding utilization and outcomes of LAAO performed in cancer patients. We investigated the utilization and short-term outcomes of this specific patient population using population-based databases of the Unites States.

## Methods

### Data resource

We queried data from National Inpatient Sample (NIS) and National Readmissions Database (NRD) for the year 2016 to 2019 from the Healthcare Cost and Utilization Project (HCUP), developed by the Agency for Healthcare Research and Quality [11, 12]. The present study was exempted from Institutional Review Board of Cook County Health as patient identifiers have been removed from HCUP datasets. We used NIS for patients’ characteristics analysis and in-hospital outcomes, and NRD for readmission measurements. Diagnosis and procedure codes were reported using International Classification of Diseases, Tenth Revision, Clinical Modification/Procedure Coding System (ICD-10-CM/PCS) in all dataset used in this study. HCUP Elixhauser Comorbidity Software was utilized for processing comorbidities [13].

### Study population

ICD-10-PCS code 02L73DK was used for identifying all LAAO procedures performed during 2016–2019. We included patients ≥ 18 years of age with primary diagnosis of AF using ICD-10-CM code I48. No additional patients were excluded for in-hospital outcome analysis using NIS databases. For readmission analysis using NRD databases, we excluded patients who died at the index hospitalization and who were discharged in the month of December as NRD databases not tracking patients across the calendar year.

### Patient and hospital characteristics

We extracted data for patient demographics (age, gender, race/ethnicity, median household income, insurance) and hospital characteristics (region, bed-size, teaching status, location). We identified comorbidities (coronary artery disease [CAD], prior cerebrovascular disease, prior coronary artery bypass grafting [CABG], heart failure, mitral valve stenosis, all valvular disease, hypertension, diabetes mellitus, obesity, chronic pulmonary disease, pulmonary circulation disorders, liver disease, renal failure, peripheral vascular disease, anemia, coagulopathy, alcohol dependent disorder, hyperthyroidism) using both HCUP Elixhauser Comorbidity Software and ICD-10-CM.

### Outcomes measured

Primary outcomes for NIS database analysis included in-hospital mortality, length of stay and cost of care. Secondary outcomes were in-hospital complications including stroke (ischemic, hemorrhagic, intra-/peri-procedure), systemic embolism, open or percutaneous pericardial drainage, other pericardial complications, major bleed and device complications. Outcomes for NRD database analysis included 30-day readmission rates and top five readmission primary diagnoses.

### Statistical analysis

We reported descriptive statistics as mean ± SD for continuous variables and n (%) for categorical variables. Data were compared using Student t test for continuous variables and chi-square for categorical variables. The variables for primary or secondary outcomes were analyzed separately in univariate logistic models and those with p value less than 0.2 were included in multivariable logistic models. Results from these models were presented as odds ratio (OR) and 95% confidence intervals (CI). We used Kaplan-Meier analysis to visualize the probability of readmission-free period after LAAO between patients with and without cancer. All analyses were performed by StataSE 17 (TX: StataCorp LLC, 2021).

## Results

### Baseline characteristics

Percutaneous LAAO procedure was performed in 60,380 patients with AF from 2016 to 2019, among which the number of patients with a diagnosis of cancer was 1,845 (3.06%) (Table 1). We found that cancer patients who underwent percutaneous LAAO procedure were older (77.26 ± 0.37 vs. 76.09 ± 0.09, *p* = 0.002). More male patients than female patients were in the cancer group (69.38% vs. 30.62%, *p* < 0.001) (Table 1). For comorbidities, patients with cancer were more prone to have renal failure (28.73% vs. 23.58%, *p* = 0.021), anemia (7.59% vs. 4.83%, *p* = 0.015) and coagulopathy (14.09% vs. 3.71%, *p* < 0.001). No significance was found regarding other comorbidities including CAD, prior cerebrovascular disease, prior CABG, heart failure, mitral valve stenosis, valvular disease, hypertension, diabetes mellitus, obesity, chronic pulmonary disease, pulmonary circulation disorders, liver disease, peripheral vascular disease, alcohol dependent disorder and hyperthyroidism (Table 1). Patients with cancer who had the percutaneous LAAO procedure tended to have higher household income (*p* < 0.001) (Table 1). We didn’t find any statistical significance in race/ethnicity, primary payer and hospital characteristics (including hospital region, hospital bed size and hospital teaching status) (Table 1).

**Table 1:** Baseline characteristics of the study population in NIS databases

Baseline characteristics	Non-cancer (%) *N* = 58,535	Cancer (%) *N* = 1845	Overall (%) *N* = 60,380	*P*-value
Age (mean ± std err)	76.09 ± 0.09	77.26 ± 0.37	76.12 ± 0.08	0.002
*Gender*				< 0.001
Male	58.06	69.38	58.40	
Female	41.94	30.62	41.60	
*Race/ethnicity*				0.300
White	87.59	86.91	87.57	
Black	4.09	4.74	4.11	
Hispanic	4.94	4.46	4.93	
Asian or pacific islander	1.36	2.51	1.39	
Native American	0.33	0.56	0.33	
Others	1.69	0.84	1.67	
*Comorbidity*				
Coronary artery disease	49.67	51.76	49.74	0.428
Prior cerebrovascular disease	27.23	22.76	27.10	0.060
Prior CABG	14.38	13.82	14.37	0.762
Heart failure	39.05	42.01	39.14	0.257
Mitral valve stenosis	0.22	0.27	0.22	0.846
Valvular disease	20.82	20.87	20.82	0.981
Hypertension	86.86	87.26	86.87	0.824
Diabetes mellitus	35.01	34.15	34.99	0.725
Obesity	16.94	16.53	16.93	0.829
Chronic pulmonary disease	21.96	18.97	21.87	0.166
Pulmonary circulation disorders	6.24	7.59	6.28	0.280
Liver disease	2.55	2.44	2.55	0.891
Renal failure	23.58	28.73	23.74	0.021
Peripheral vascular disease	16.28	17.62	16.32	0.489
Anemia	4.83	7.59	4.91	0.015
Coagulopathy	3.71	14.09	4.02	< 0.001
Alcohol dependent disorder	1.38	1.90	1.39	0.394
Hyperthyroidism	0.43	0.54	0.43	0.740
*Median household income*				0.001
0-25th percentile	21.77	15.85	21.59	
26-50th percentile	25.92	24.04	25.86	
51-75th percentile	27.96	27.05	27.94	
76-100th percentile	24.35	33.06	24.62	
*Primary payer*				0.453
Medicare/Medicaid	90.01	91.28	90.05	
Private insurance	7.89	7.90	7.89	
Self-pay	0.47	0.00	0.46	
Other	1.62	0.82	1.60	
*Hospital characteristics*				
Hospital region				0.138
Northeast	16.47	20.05	16.58	
Midwest	22.49	21.14	22.45	
South	39.22	34.42	39.07	
West	21.82	24.39	21.90	
Hospital bed size				0.624
Small	10.61	10.30	10.60	
Medium	23.11	21.14	23.05	
Large	66.29	68.56	66.35	
Hospital teaching status				0.490
Non-teaching	11.81	10.57	11.78	
Teaching	88.19	89.43	88.22	
Hospital location				0.044
Urban	97.99	99.46	98.04	
Rural	2.01	0.54	1.96	

### In-hospital outcomes and complications

Patient population with cancer had similar in-hospital mortality rate compared with those without cancer (0.27% vs. 0.15%, *p* = 0.54), and the costs of care were similar amount (121,510.2 ± 4,462.83 USD vs. 119,254.9 ± 1,993.101 USD, *p* = 0.561) (Table 2). However, cancer patients who underwent the procedure had longer length of stay for the indicated hospitalization (1.59 ± 0.11 days vs. 1.32 ± 0.02 days, *p* = 0.013), and there was a trend that patients with cancer were less likely to be discharged on the same day, though there was no statistical significance (length of stay > 1 day: 15.99% vs. 12.85%, *p* = 0.07) (Table 2).

**Table 2:** In-hospital outcomes and complications of the study population in NIS databases

	Non-cancer (%) *N* = 58,535	Cancer (%) *N* = 1845	Overall (%) *N* = 60,380	*P*-value
In-hospital outcomes				
In-hospital mortality	0.15	0.27	0.15	0.537
Length of stay (mean ± std err) (days)	1.32 ± 0.02	1.59 ± 0.11	1.33 ± 0.02	0.013
Length of stay > 1 day	12.85	15.99	12.94	0.068
Cost of care (mean ± std err) (USD)	119254.9 ± 1993.1	121510.2 ± 4462.8	119324.2 ± 2003.6	0.561
**In-hospital complications**				
Ischemic stroke	0.16	0.27	0.17	0.614
Hemorrhagic stroke	0.08	0.00	0.08	0.591
Intra-/peri-procedure stroke	0.04	0.00	0.04	0.690
Systemic embolism	0.09	0.00	0.09	0.554
Open/percutaneous pericardial drainage	1.12	2.44	1.16	0.020
Other pericardial complications	0.73	1.63	0.76	0.055
Major bleed^a^	0.14	1.15	0.18	< 0.001
Device complications	0.17	0.27	0.17	0.633

^a^Major bleed: Intracranial or gastrointestinal bleeding required blood product transfusion

**Fig. 1:**
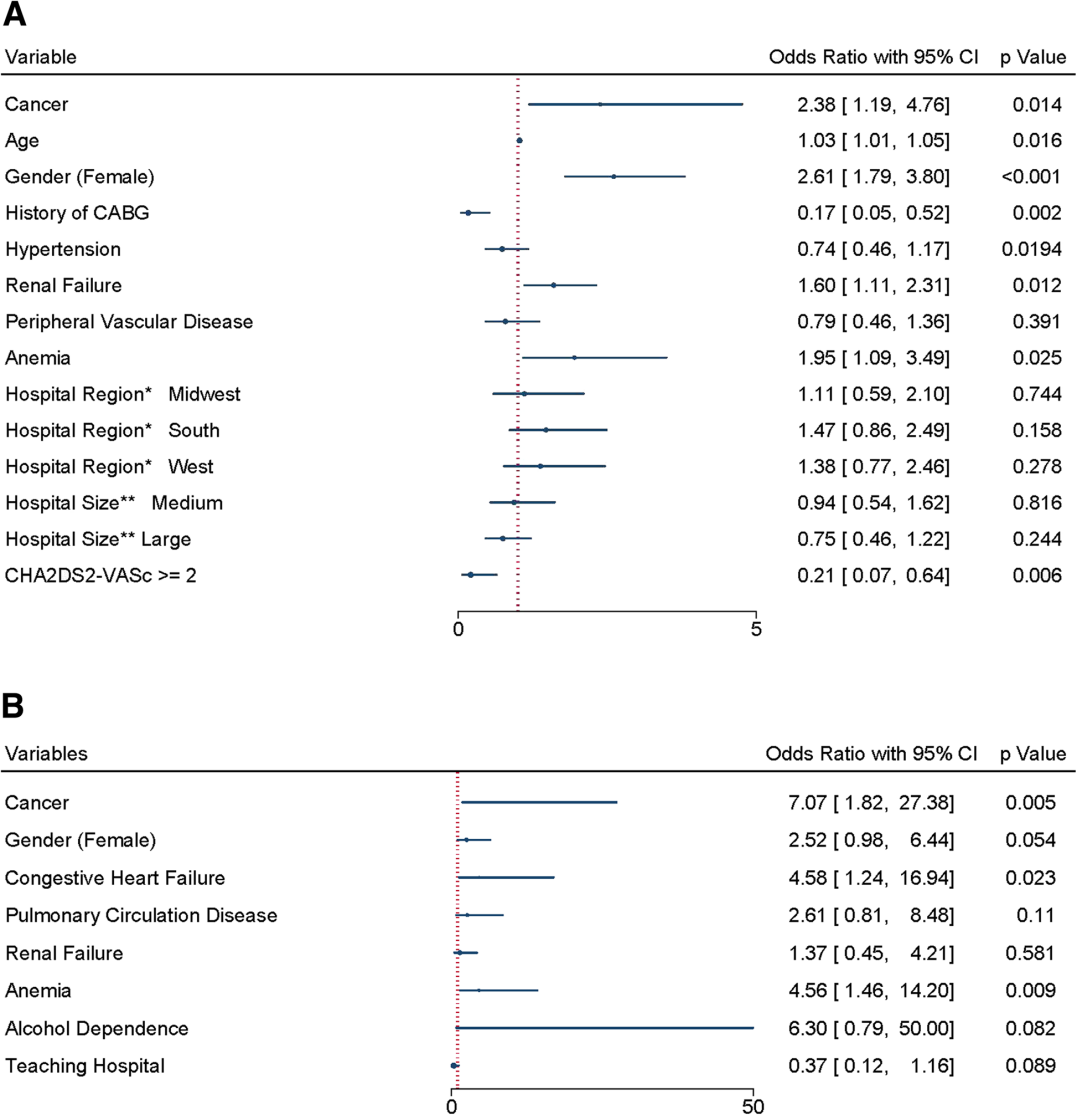
**A**, multivariate logistic regression result for open/percutaneous pericardial drainage events; **B**, multivariate logistic regression result for major bleeding events

We found that patient with cancer who underwent the procedure had more incidences of open or percutaneous pericardial drainage (2.44% vs. 1.12%, *p* = 0.020) and also major bleeding events defined as intracranial or gastrointestinal bleeding required blood product transfusion (1.15% vs. 0.14%, *p* < 0.001) (Table 2). Other in-hospital complications occurrences were not found to be significantly different, including ischemic stroke, hemorrhagic stroke, intra or peri-procedure stroke, systemic embolism, other pericardial complications and device complications (Table 2). After multivariable logistic regression, patient with cancer was an independent risk factor for open or percutaneous pericardial drainage (aOR 2.38; 95% CI 1.19–4.76) (Fig. 1A) and major bleeding events (aOR 7.07; 95% CI 1.82–27.38) (Fig. 1B).

### Readmission analysis

**Fig. 2:**
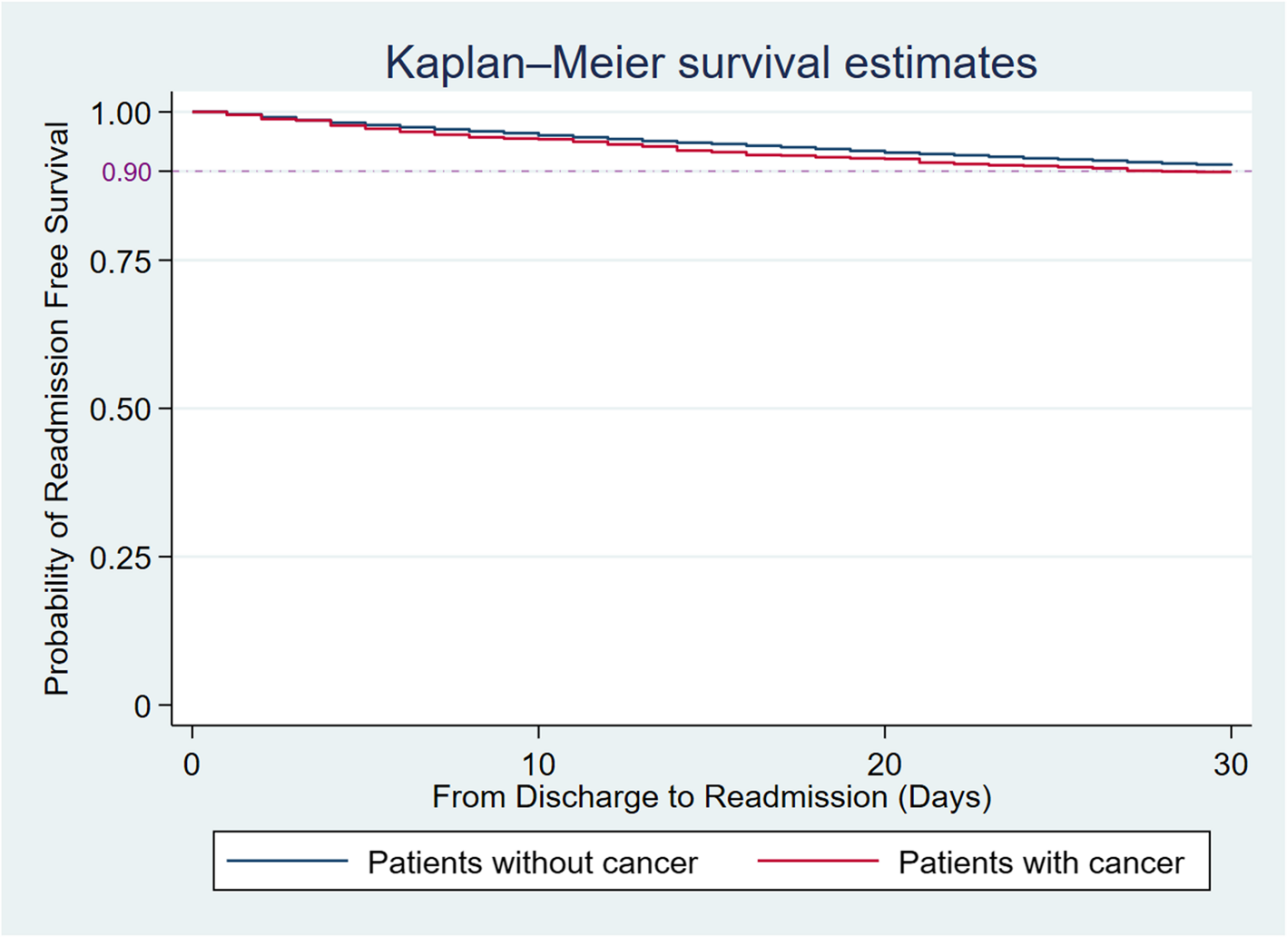
Probability of readmission-free survival period from discharge to readmission between patients without cancer and patient with cancer groups (blue: patients without cancer group; red: patients with cancer group)

A total of 49,882 index hospitalizations for LAAO were identified from NRD 2016–2019, among which 1545 (3.1%) patients had a secondary diagnosis of cancer. The overall readmission rate of LAAO patients was 9.1%. There was no statistical significance of 30-day readmission rate between patients with and without cancer (10.0% vs. 9.1%, *p* = 0.34) (Fig. 2).

The most common primary diagnoses for readmission among cancer patients were GI hemorrhage (ICD-10-CM K922), unspecified atrial fibrillation (ICD-10-CM I4891), non-ST elevation myocardial infarction (ICD-10-CM I214), pneumonia (ICD-10-CM J189), chronic obstructive pulmonary disease with (acute) exacerbation (ICD-10-CM J441) (Table 3). The most common primary diagnosis for readmission in patients without cancer were hypertensive heart and chronic kidney disease (ICD-10-CM I130), sepsis (ICD-10-CM A419), GI hemorrhage (ICD-10-CM K922), angiodysplasia of stomach and duodenum with bleeding (ICD-10-CM K31811), paroxysmal atrial fibrillation (ICD-10-CM I480) (Table 3).

**Table 3:** The five most common primary diagnoses of readmission

Non-cancer group		Cancer group	
ICD-10-CM	Diagnosis	ICD-10-CM	Diagnosis
I130	Hypertensive heart and chronic kidney disease	K922	GI hemorrhage
A419	Sepsis	I4891	Unspecified atrial fibrillation
K922	GI hemorrhage	I214	Non-ST elevation myocardial infarction
K31811	Angiodysplasia of stomach and duodenum with bleeding	J189	Pneumonia
I480	Paroxysmal atrial fibrillation	J441	Chronic obstructive pulmonary disease with (acute) exacerbation

## Discussion

Percutaneous LAAO procedure has been increasingly conducted since its approval [14]. We can predict that cancer patient will become an increasing patient population who will benefit from percutaneous LAAO procedure to avoid risk of bleeding from anticoagulation for AF, as the prognosis of certain cancer types has been boosted by evolving cancer therapies. From our study, we found that among patients who had the percutaneous LAAO procedure, cancer patients were older, more male patients and had more comorbidities such as renal failure, anemia and coagulopathy, of which anemia was also found to be more prevalent in a previous study [15]. It is possible that anemia is more prevalent in cancer patients [16] and we included hematological malignancy in our analysis, so it becomes a more common contraindication for anticoagulation in patients who developed AF, which might prompt patients and physicians to choose percutaneous LAAO as an alternative.

There are conflicting evidences about whether cancer is a risk factor of in-hospital mortality after percutaneous LAAO procedure [15, 17], while our study showed there was no statistical significance. Additionally, we showed the costs for indicated hospitalization were also similar between two patient groups. However, we did find that patients with cancer required longer hospital stay, and there was a trend that cancer patients were less likely to be discharged same day after the procedure, which might be due to more complications. Pericardial effusion is one of the most common complications after percutaneous LAAO procedure, rate from 0.68% to 3.1% in previous studies [14, 18]. Our study showed that cancer was an independent risk factor for pericardial effusion requiring open or percutaneous pericardial drainage. Major bleeding events that was defined as intracranial or gastrointestinal bleeding requiring blood products transfusion was also identified as a significant complication for cancer patients who underwent percutaneous LAAO procedure, which was rarely demonstrated from previous studies. This might be correlated that coagulopathy as a comorbidity was significantly higher in the cancer patient population that we investigated. Interestingly, our results did not show any differences in in-hospital ischemic comorbidities, such as ischemic stroke and systemic embolism, which was different from a recent study [15]; a recent published study also demonstrated that there was no significant difference in the rate of combined stroke between cancer and noncancer patients in a 4-year follow up cohort [19].

Overall, we concluded that percutaneous LAAO procedure is relatively safe in cancer patients with AF and contraindication to anticoagulation. However, more attention is needed regarding complications such as pericardial effusion and bleeding events, which might be culprit for in-hospital mortality and longer or complicated hospital stays.

Our study has limitations inherent to the data source, which lacks information of clinical course of cancers as the status of the cancer history cannot be differentiated between active cancer patients or cancer survivors. Data of specific treatments were not provided in the databases, such as antithrombotic therapy and cancer-specific therapies, of which certain drugs can lead to increased risk of thrombotic or bleeding consequences. More cases and longer observation time are needed to assist with balancing risks and benefits in cancer patients complicated by AF who might qualify for percutaneous LAAO procedure.

## Conclusions

We were among the first researchers who investigated the utilization of percutaneous LAAO procedure in cancer patients in a real-world cohort. From our results, cancer patients had similar in-hospital outcomes after percutaneous LAAO procedure comparing to patients without cancer as a secondary diagnosis, in terms of in-hospital mortality, total hospital charges, in-hospital complications including intra-/post-procedural stroke or systemic embolism; but cancer patients had longer hospital stay, required greater number of interventions for pericardial effusion and tended to have more major bleeding events. Overall, percutaneous LAAO is a promising procedure in cancer patients complicated by AF with contraindication to anticoagulation, but more cases and longer observation time need to be conducted to assist with balancing risks and benefits and minimizing complications in this specific patient population.

### Clinical perspectives

We investigated the utilization of percutaneous LAAO procedure in cancer patients in a real-world cohort. We demonstrated that percutaneous LAAO is a promising procedure in cancer patients complicated by AF with contraindication to anticoagulation.

## Data Availability

Data were extracted from National Inpatient Sample (NIS) and National Readmissions Database (NRD) for the year 2016 to 2019 from the Healthcare Cost and Utilization Project (HCUP), developed by the Agency for Healthcare Research and Quality, which can be assessed through websites www.hcup-us.ahrq.gov/nisoverview.jsp, www.hcup-us.ahrq.gov/nrdoverview.jsp.

## Abbreviations

LAAO: Left atrial appendage occlusion
aOR: Adjusted odds ratio
CI: Confidence interval
AF: Atrial fibrillation/flutter
NIS: National Inpatient Sample
NRD: National Readmissions Database
HCUP: Healthcare Cost and Utilization Project
ICD-10-CM/PCS: International Classification of Diseases, Tenth Revision, Clinical Modification/Procedure Coding System
CAD: Coronary artery disease
CABG: Coronary artery bypass grafting
